# Intra-specific Variation in the Social Behavior of Barbary macaques (*Macaca sylvanus*)

**DOI:** 10.3389/fpsyg.2021.666166

**Published:** 2021-10-13

**Authors:** Federica Amici, Anja Widdig, Lorenzo von Fersen, Alvaro Lopez Caicoya, Bonaventura Majolo

**Affiliations:** ^1^Department of Human Behavior, Ecology and Culture, Research Group “Primate Behavioural Ecology”, Max Planck Institute for Evolutionary Anthropology, Leipzig, Germany; ^2^Faculty of Life Science, Behavioral Ecology Research Group, Institute of Biology, University of Leipzig, Leipzig, Germany; ^3^Zoo Nuernberg, Nuernberg, Germany; ^4^Department of Clinical Psychology and Psychobiology, Faculty of Psychology, University of Barcelona, Barcelona, Spain; ^5^Institute of Neurosciences, University of Barcelona, Barcelona, Spain; ^6^School of Psychology, University of Lincoln, Lincoln, United Kingdom

**Keywords:** intra-specific variation, Barbary macaques, neophobia, social integration, access to food, social behavior

## Abstract

Non-human primates show an impressive behavioral diversity, both across and within species. However, the factors explaining intra-specific behavioral variation across groups and individuals are yet understudied. Here, we aimed to assess how group size and living conditions (i.e., captive, semi-free-ranging, wild) are linked to behavioral variation in 5 groups of Barbary macaques (N=137 individuals). In each group, we collected observational data on the time individuals spent in social interactions and on the group dominance style, along with experimental data on social tolerance over food and neophobia. Our results showed that differences in group size predicted differences in the time spent in social interactions, with smaller groups spending a higher proportion of time in close spatial proximity, but a lower proportion of time grooming. Moreover, group size predicted variation in dominance style, with smaller groups being more despotic. Social tolerance was affected by both group size and living conditions, being higher in smaller groups and in groups living in less natural conditions. Finally, individual characteristics also explained variation in social tolerance and neophobia, with socially integrated individuals having higher access to food sources, and higher-ranking ones being more neophobic. Overall, our results support the view that intra-specific variation is a crucial aspect in primate social behavior and call for more comparative studies to better understand the sources of within-species variation.

## Introduction

Non-human primates (hereafter, primates) show a high degree of behavioral diversity in terms of ecology, sociality, and cognition, not only across species (Mitani et al., 2012), but also within the same species (Struhsaker, 2000; Strier, 2003, 2016). Intra-specific variation can happen at various levels, including differences (i) across conspecific groups and populations and (ii) across individuals of the same group (hereafter, inter-individual variation; Strier, 2016). To date, inter-individual variation in primate behavior has been the focus of abundant research; it has shown how individuals within the same group may use different behavioral strategies depending on their age, dominance rank, sex, or personality (e.g., Hosey, 2005; Lehmann and Boesch, 2008, 2009; Slater et al., 2009; Lonsdorf et al., 2014; Kulik et al., 2015a,b). Higher-ranking individuals, for instance, are usually involved in social interactions more often than lower-ranking ones (e.g., Seyfarth, 1977; Schino, 2001; Tiddi et al., 2012), and they usually have priority of access to limited resources (e.g., Dunbar and Dunbar, 1977; Janson and van Schaik, 1988; Hohmann et al., 2006; King et al., 2008; Romero and Castellanos, 2010) and may thus gain lower potential payoffs from novelty, being more likely to fearfully respond to it (see Laland and Reader, 1999; Reader and Laland, 2001, 2003; Amici et al., 2020).

In contrast, much less is known about variation across conspecific groups (see Strier, 2016; see Kappeler and Kraus, 2010; Schradin, 2013). Two socio-ecological factors that may cause significant intra-specific variation in primate behavior are group size and living conditions. Group size may vary depending on the ecological conditions (e.g., resource availability or predation risk: e.g., van Schaik, 1983; Janson and Goldsmith, 1995) and might importantly affect several aspects of primate behavior, from feeding ecology (e.g., Wrangham et al., 1993; Janson and Goldsmith, 1995), to sociality (Sterck et al., 1997; Lehmann et al., 2007) and cognition (Deaner et al., 2000; MacLean et al., 2013; Sandel et al., 2016). First, group size may affect the time spent in social interactions within the group (Japanese macaques, *Macaca fuscata*: Majolo et al., 2009). On the one hand, larger groups may spend less time in grooming interactions, either because they face stronger competition for social partners, or because they may need to travel longer to forage and have less time for social interactions. On the other hand, larger groups may offer a larger availability of potential social partners, so that individuals may more easily find a suitable conspecific to interact with (see Majolo et al., 2008, across primates, and Majolo et al., 2009, on Japanese macaques). Previous studies have shown that larger groups spend more time grooming as compared to smaller groups (across primates: Dunbar, 1992; Lehmann et al., 2007; in baboons, *Papio cynocephalus ursinus*: Henzi et al., 1997), likely because they have a higher number of group members with whom they need to interact to maintain social cohesiveness (Lehmann et al., 2007; see Majolo et al., 2008). Second, group size may affect dominance style. Group-living primate species have different dominance styles that vary from more to less despotic (macaques, *Macaca* spp.: Thierry et al., 2008). Less despotic species are usually characterized by more symmetrical agonistic interactions than more despotic ones, by a higher rate of counter-aggression and undecided outcomes after conflicts, by shallower dominance hierarchies and by a higher reconciliation rate (macaques: de Waal, 1989; de Waal and Luttrell, 1989; Aureli et al., 1997; Thierry, 2000, 2007; Flack and de Waal, 2004; Thierry et al., 2004; Balasubramaniam et al., 2012). In very large groups, however, higher-ranking individuals may not manage to intervene in all conflicts, and they may less successfully maintain linear hierarchies, with an increase in counter-aggression rate and undecided relationships that could result in shallower dominance hierarchies and less despotic dominance styles (in Japanese macaques, see Mori, 1977; Zhang and Watanabe, 2014). Third, social group size may affect tolerance over food sources. On the one hand, larger groups might face higher within-group food competition, leading to a significant decrease in tolerance over food sources (across primates: Janson and van Schaik, 1988; Isbell, 1991). On the other hand, larger groups may experience less between-group food competition by better monopolizing richer food patches than smaller groups (Wrangham, 1980; Janson and van Schaik, 1988), although this may not compensate the costs of higher within-group competition (Majolo et al., 2008). Moreover, higher-ranking individuals in larger groups may more likely fail to monopolize access to resources because there are too many competitors, so that more individuals may get a share of resources by using different strategies (macaques: Gomez-Melara et al., 2021). Fourth, social group size may affect the degree to which individuals avoid novelty (i.e., expressing neophobia; Greenberg and Mettke-Hofmann, 2001). In larger groups, individuals are usually less vulnerable to predation and have more opportunities of social facilitation when exposed to novelty, which may result in lower neophobia levels (e.g., capuchin monkeys, *Sapajus apella*: Visalberghi and Addessi, 2000; Addessi and Visalberghi, 2001). Several studies have indeed shown a link between larger group size and lower levels of neophobia (e.g., chimpanzees, *Pan troglodytes*: Lonsdorf, 2006; Japanese macaques: Tarnaud and Yamagiwa, 2008; capuchin monkeys: Visalberghi and Addessi, 2000). Therefore, group size may predict behavioral differences across conspecific groups in terms of social interactions, dominance style, social tolerance, and neophobia.

Similarly, living conditions may cause important intra-specific variation in primate behavior. For primates, living conditions vary along a continuum from more natural to less natural ones (Carlstead, 1996; Chang et al., 1999), depending on a variety of factors (e.g., freedom of movement, predation pressure, reliance on food provisioning by humans). In captive groups, individuals usually have high food availability, no predation risk but limited freedom of movement, and these atypical socio-ecological conditions may result in different behavioral patterns as compared to their wild conspecifics (e.g., Majolo et al., 2005; Boesch, 2007). This has raised concerns on the fact that captive individuals may not be good representatives of their wild counterparts, and that studies based on data collected in captivity may not allow reliable comparisons across species (for different perspectives on this issue, see Boesch, 2007; Tomasello and Call, 2008). First, living conditions may affect the frequency of social interactions across individuals. In captivity, individuals are usually fully provisioned by humans, and they do not need to invest time in foraging and may devote more time to social interactions, as compared to wild conspecifics (e.g., Carlstead, 1996, on mammals). Second, dominance styles are at least partially acquired through development (macaques: de Waal and Johanowicz, 1993), so that contingent living conditions may importantly affect the dominance style of a group. In captivity, the number of hiding places is limited, and higher-ranking individuals may exert more control over lower-ranking ones, with aggressive encounters becoming riskier and dominance style more despotic (bonobos, *Pan paniscus*: Stevens et al., 2007). Third, captivity may be linked to increased social tolerance over food. Being food provisioned, captive groups face little food competition, which may lead to a general increase in tolerance over food (across primates: Janson and van Schaik, 1988; Isbell, 1991). Finally, living conditions may be linked to differences in neophobia. Captive individuals are continuously exposed to human artifacts and novel objects (e.g., *via* environmental enrichment), which may result in lower levels of neophobia (e.g., baboons, *Papio ursinus*, and geladas, *Theropithecus gelada*: Bergman and Kitchen, 2009; vervet monkeys, *Chlorocebus aethiops*: van de Waal and Bshary, 2010). Therefore, different living conditions may be linked to variation across groups in terms of social interactions, dominance style, social tolerance, and neophobia.

In this study, we aimed to assess how group size and living conditions contribute to intra-specific variation in primate behavior. We combined behavioral observations and experiments to analyze social interactions (i.e., time spent in close spatial proximity or being involved in grooming interactions), dominance style, tolerance over food, and neophobia with regard to food across five groups of Barbary macaques living in different conditions. Barbary macaques live in multi-male multi-female groups, in which males migrate and females remain in their natal groups organized in matrilines. The dominance style of female Barbary macaques is considered as being relatively tolerant (Thierry, 2000). We predicted (Table 1) that larger groups, compared to smaller ones, would spend more time in close spatial proximity and grooming interactions (prediction 1a) and show less despotic dominance styles (prediction 1b), higher tolerance levels (prediction 1c), and lower neophobia (prediction 1d). Moreover, we predicted that groups living in less natural living conditions, as compared to those living in more natural conditions, would spend more time in close spatial proximity and grooming interactions (prediction 2a) and show more despotic dominance styles (prediction 2b), higher tolerance levels (prediction 2c), and lower neophobia (prediction 2d).

**Table 1:** Predictions of our study, model in which they were tested and whether they were supported by our data.

Prediction		Model	Support
1. Larger groups…			
a.	… spend *more* time in (i) close spatial proximity and (ii) grooming	1–2	No–Yes
b.	… have *less* despotic dominance styles	-	Yes
c.	… have *higher* tolerance over food	3	No
d.	… are *less* neophobic	5	No
2. Groups living in less natural conditions….			
a.	… spend *more* time in (i) close spatial proximity and (ii) grooming	1–2	No–No
b.	… have *more* despotic dominance styles	-	No
c.	… have *higher* tolerance over food	3	Yes
d.	… are *less* neophobic	5	No

## Materials and Methods

### Subjects

We worked on five different groups living in conditions: one wild group, two semi-free-ranging groups, and two captive groups. All groups included males and females of different age classes and dominance ranks (*N*=137 individuals, excluding infants; see Table 2), who were individually recognized based on their external appearance (e.g., size, color, fur length, and scars) and partially on markings (in the two semi-free-ranging groups). The wild group (*N*=20) lived in the Gibraltar Nature Reserve (36°08′37.5"N 5°20′36.5"W), in an area with steep cliffs and sparse vegetation, in a military zone which cannot be accessed by local inhabitants and tourists. The study groups mostly foraged and fed on natural food, but they were also partially provisioned with small quantities of fruit and vegetables on a daily basis by a local NGO. They were completely free to move in their natural environment and were not protected from predators. Therefore, we considered this group to live in natural conditions and refer to them as being “wild.” We further studied two semi-free-ranging groups in Kintzheim, in the park La Montagne des Singes. The first group (Kintzheim-1, *N*=65) lived in an enclosure of 7ha with trees and natural vegetation, in which they could move with no restrictions. The group had always access to fresh water and fed on natural vegetation, but it was also provided once a day with vegetables, fruit, pellets, and wheat by the park staff. This group was not visited by tourists. The second group (Kintzheim-2, *N*=56) lived in an enclosure of 11ha with trees and natural vegetation, which they shared with another group of Barbary macaques that was not observed in this study. The two groups could freely move in their enclosure, but they rarely interacted because they occupied different areas. This group was regularly visited by tourists, who could feed the monkeys along touristic trails with unsweetened popcorn provided at the park entrance. As for the other group, the monkeys had access to freshwater and fed on natural vegetation, but they were also provided food by the park staff three times a day to ensure sufficient food intake for all individuals independent of rank and age. Given the intermediate characteristics of the living conditions of these two groups (in terms of food provisioning, freedom of movement, and predation pressure), we refer to them in this study as to the “semi-free-ranging” ones. Finally, we tested two captive groups living in the Cordoba zoo (*N*=7) and in the Nürnberg zoo (*N*=5). In both zoos, monkeys had access to indoor areas with several enrichment activities, and outdoor enclosures with trees and natural vegetation. In both groups, the monkeys were fed once a day by the keepers with vegetables, fruit, and pellets, while water was always available. All groups were habituated to the experimenter and routinely exposed to humans (i.e., the wild group in Gibraltar to NGO workers, the semi-free-ranging group in Kintzheim-1 to keepers and workers of the park, and the other groups to keepers, workers, and visitors).

**Table 2:** Study groups (including the individuals who died during the study period but participated in the tasks), their living conditions, location, number of individuals (plus infants, i.e., individuals younger than 1year), number of individuals for each age class (i.e., juveniles: females younger than 3, and males younger than 4; subadults: females younger than 4, and males younger than 5; and adults: females from 4years, and males from 5years), and sex (females and males).

Living conditions	Location	Group size (+ infants)	Juveniles–Subadults–Adults	Females–Males
Captive	Cordoba	6 (+1)	1 - 0 - 5	4 - 2
	Nürnberg	5 (+0)	0 - 0 - 5	5 - 0
Semi-free-ranging	Kintzheim-1	59 (+6)	2 - 0 - 57	33 - 26
	Kintzheim-2	48 (+8)	6 - 4 - 38	28 - 20
Wild	Gibraltar	19 (+1)	2 - 0 - 17	10 - 9

### Ethics

All experimental protocols were approved by the ethics committees of the Helping Hand Trust in Gibraltar, La Montagne des Singes in France (where we worked with two groups), the Cordoba zoo in Spain, and the Nürnberg zoo in Germany. No further permits were required. The study was mainly observational, and all study groups were used to receiving food from humans (see below for details). Individuals participated in the experimental tasks on a completely voluntary basis, and they were never food or water deprived to facilitate participation. The study was carried out in accordance with the national regulations of all the countries in which the study was conducted.

### Behavioral Observations

In each group, we collected information using 20-min focal samples (Altmann, 1974), distributing them throughout the day (i.e., from 7:30 to 19:30). During focal samples, we recorded the exact duration of all grooming interactions in which the focal subject was involved, and (at 30-s intervals) whether the focal subject was in close spatial proximity (i.e., body contact or 2m proximity) with another partner or socially playing with another group member. The order of the focal subjects was determined with a random permutation procedure, ensuring at least 60 min between focal samples on the same individual. We conducted 177 focals in Gibraltar (on average, 10 20-min focal per subject, ranging from 5 to 18), 1,241 in Kintzheim-1 (on average, 22 focals per subject, ranging from 3 to 33), 1,097 in Kintzheim-2 (on average, 26 per subject, ranging from 12 to 30), 128 in Cordoba (on average, 26 focals per subject, ranging from 25 to 26), and 122 in Nürnberg (on average, 24 focals per subject, ranging from 24 to 25). For each individual, we then assessed (i) the proportion of time spent in close spatial proximity with other group members (as the proportion of observations in which the subject was in close spatial proximity with a partner, out of the observations in which the focal subject was visible); (ii) the proportion of time spent playing with other group members (as the proportion of observations in which the subject was playing with a partner, out of the observations in which the focal subject was visible); and (iii) the proportion of time spent in grooming interactions (as the time spent in grooming interactions, out of the total time in which the subject was visible).

We further used the all-occurrences method (Altmann, 1974) to record all dyadic agonistic interactions and determine the individual ranks, the steepness of the hierarchy, and the proportion of agonistic interactions against the hierarchy (i.e., interactions were the aggressor is an animal ranking lower than the target of aggression; Young and Isbell, 2002; de Vries et al., 2006; see below). Finally, we collected group scans on an hourly basis (Altmann, 1974), recording the spatially closest partner for each individual. We then used this measure to assess the social network and the individual centralities (as a measure of social integration; Farine, 2017; see below).

We observed all group members except for infants (i.e., individuals younger than 1year old). Only in focal observations, we also excluded juveniles (i.e., females between 1 and 3, and males between 1 and 4) from the observations. In Kintzheim-1, four individuals died in 2017, so that less data are available for them; in Kintzheim-2, one individual was never observed being involved in any agonistic interaction, so that no rank could be determined for this individual. Observations took place in Gibraltar from October 2017 to March 2018; in Kintzheim-1 from September 2016 to June 2017; in Kintzheim-2 from March to June 2017; in Cordoba in July 2017; and in Nürnberg from January to February 2017. As four different researchers conducted behavioral observations in the groups, we ensured inter-observer reliability in that researchers only started collecting data independently after reaching a reliability higher than 90% with the trainer (Kaufman and Rosenthal, 2009).

### Experimental Setting

In each group, we further administered one social tolerance and one neophobia task each. We tested individuals in a 4m×4m square flat area with little to no vegetation (hereafter: testing area) that was divided into 4 identical 1m×1m squares marked by stones or branches. The testing area was located in an area where the group usually foraged, to ensure the participation of individuals in the group. Experiments were mostly conducted in the morning, before feeding took place, to increase motivation in all the study groups.

In the social tolerance task, we aimed to assess social tolerance over food. During the task, the experimenter threw banana slices in the testing area (see Amici et al., 2020). In the Nürnberg zoo, the experimenter had access to the enclosure, while in the Cordoba zoo the food was thrown from an area which was not accessible to the macaques. Food pieces were proportional to the number of adults in each group (i.e., 8 food pieces in Gibraltar, 28 food pieces in Kintzheim-1, 22 in Kintzheim-2, and 4 in Cordoba and Nürnberg) and were distributed throughout the testing area. Sessions started when the first macaque entered the testing area and ended when the last food piece was retrieved, or if no monkey was in the testing area or retrieved food for more than 30s. As the session was over, the experimenter cleaned the testing area and waited at least 5min before starting a new session. Each group was tested for 60 sessions (except for Kintzheim-1, where only 59 sessions were administered), on different testing days (i.e., 7days in Gibraltar and Kintzheim-1, 6days in Kintzheim-2, and 3 in Cordoba and Nürnberg). The majority of the monkeys (i.e., 72%) participated in at least one session of the social tolerance task (i.e., 14/19 macaques in Gibraltar, 38/59 in Kintzheim-1, 38/48 in Kintzheim-2, 5/6 in Cordoba, and 4/5 in Nürnberg).

In the neophobia task, we used two conditions (i.e., food and object conditions) to assess how individuals react to novelty in different contexts (see Amici et al., 2020). We followed exactly the same procedure as in the social tolerance task, except that (i) in the food condition, half of the banana pieces had been dyed with a novel food color (i.e., red or blue) having no odor and no taste; (ii) in the object condition, half of the banana pieces were distributed in two non-adjacent 2m×2m squares of the testing area previously covered with local familiar leaves, and half in the two other 2m×2m squares previously covered with novel objects (i.e., novel pieces of salt dough with leaf shape and size, colored in yellow or silver). In both food and object conditions, half of the food pieces were familiar banana slices as in the social tolerance task, so that novel and familiar stimuli were simultaneously present. Each group was tested for 20 sessions in the food condition (with red novel stimuli), followed by 20 sessions in the object condition (with yellow novel stimuli), 20 further sessions in the food condition (with blue novel stimuli), and 20 sessions in the object condition (with silver novel stimuli), on different testing days (i.e., 8days in Gibraltar, Kintzheim-1, and Kintzheim-2, and 4 in Cordoba and Nürnberg). The majority of the monkeys (i.e., 69%) participated in at least one session of the neophobia task (i.e., 19/19 macaques in Gibraltar, 35/59 in Kintzheim-1, 32/48 in Kintzheim-2, 5/6 in Cordoba, and 4/5 in Nürnberg).

We video-recorded all sessions, recording the name of each individual entering and/or moving in the testing area, so that we could later extract the following data from the videos: (i) how many food pieces were eaten by each individual per session; (ii) whether the food retrieved was familiar/novel (in the food condition) or collected in the squares with familiar/novel objects (in the object condition); and (iii) how much familiar/novel food was still available in the testing area whenever subjects retrieved a piece of food. The number of food pieces retrieved in each session did not always correspond to the number of pieces in the testing area, either because the same piece was taken by more than one monkey (e.g., because it fell and got broken), or because some pieces were not collected (e.g., if they were inadvertently covered). Infants only seldom retrieved food, and as we collected no social information on them, we did not code these trials.

### Data Analysis

In order to assess the individual ranks, we used the Elo methods (EloRating package, version 0.43; Neumann et al., 2011) and analyzed all interactions with a clear winner–loser outcome (i.e., 125 interactions in Gibraltar, 1,412 in Kintzheim-1, 1,253 in Kintzheim-2, 229 in Cordoba, and 64 in Nürnberg). The Elo values obtained (setting the k factor at 100 and the starting values at 1000) were then averaged in each group over the study period and then standardized (so that 0 corresponded to the lowest rank, and 1 to the highest one). As the Elo ranks were very stable in all groups over the whole study period (i.e., 0.992 in Gibraltar, 0.989 in Kintzheim-1, 0.925 in Kintzheim-2, 0.923 in Cordoba, and 0.931 in Nürnberg; see Neumann and Kulik, 2020), we included no burn-in periods.

For each group, we also used the package steepness (version 0.2-2 10; Leiva and de Vries, 2014) in R (R Core Team, 2020) to assess the steepness of the hierarchy, which is a crucial aspect of dominance style (Thierry, 2000; Balasubramaniam et al., 2012). The steepness of the hierarchy was calculated as the absolute value of the slope straight line fitted to the normalized David’s scores, which were assessed on the basis of proportions of wins of dyadic agonistic interactions (and which strongly correlated with the Elo ranks, as assessed with exact Spearman’s correlations: *r*=0.954, *p* < 0.001 in Gibraltar; *r*=0.926, *p* < 0.001in Kintzheim-1; *r*=0.879, *p* < 0.001 in Kintzheim-2; *r*=0.943, *p* = 0.017 in Cordoba; and *r*=1.000, *p* = 0.017 in Nürnberg; see de Vries et al., 2006). However, the steepness of the hierarchy decreases when the number of unknown relationships is higher (Klass and Cords, 2011), and the proportion of unknown relationships varied across our study groups (i.e., 57% of the 171 possible dyads were unknown in Gibraltar, 59% of the 1,653 possible dyads in Kintzheim-1, 67% of the 1,081 possible dyads in Kintzheim-2, 0% of the 15 possible dyads in Cordoba, and 0% of the 10 possible dyads in Nürnberg). Therefore, we randomly removed dyads with known relationship from all the groups but Kintzheim-2 (which had the highest proportion), to reach the same proportion of unknown relationships in all groups (i.e., 67%). We then averaged the values obtained over 1,000 iterations to obtain adjusted steepness values for each group (see Klass and Cords, 2011; Beltrán Francés et al., 2020).

In order to assess the individual centralities and the social networks, we analyzed the group scans conducted in each group (i.e., 47 group scans in Gibraltar, 393 in Kintzheim-1, 389 in Kintzheim-2, 163 in Cordoba, and 148 in Nürnberg). The number of scans was lower for the wild group in Gibraltar, because the group lived on a cliff, largely inaccessible to the experimenter, and it was therefore more often out of view. From these measures, we constructed an undirected weighted matrix which we analyzed in R (R Core Team, 2020) with the packages vegan 2.5-3 (Oksanen et al., 2018), asnipe 1.1.10 (Farine, 2018), and igraph 1.2.1 (Csardi and Nepusz, 2006). The individual values we obtained assessed eigenvector centrality as the sum of the centralities of an individual’s neighbors, which varied from 0 to 1 (i.e., the value 0 being assigned to the least socially integrated individuals; Farine and Whitehead, 2015; Farine, 2017).

In order to assess intra-specific variation in terms of social interactions, social tolerance, and neophobia, we prepared four data-sets and then used generalized linear mixed models (Baayen et al., 2008) with the glmmTMB package (version 1.0.16; Brooks et al., 2017) in R (R Core Team, 2020). The dataset for Models 1 and 2 allowed assessing variation in social interactions across the study groups. In this dataset, we entered one data point for each group member (only excluding infants; N = 137) specifying the group the individual belonged to (i.e., Gibraltar, Kintzheim-1 or 2, Cordoba, or Nürnberg), group size, living conditions (i.e., from 1 to 3, with 1 indicating less natural and 3 more natural living conditions), sex, age class, rank, and centrality, and the proportion of time spent in close spatial proximity or in grooming interactions (see above). We used two generalized linear mixed models to assess whether the proportion of time spent in close spatial proximity (Model 1) or in grooming interactions (Model 2) varied depending on their living conditions and/or their group size (i.e., including living conditions and group size as test predictors), when controlling for individuals’ sex, age class, rank, and centrality, including group identity as random factor. As the responses were proportions (including 0 and 1 s), we ran the model with a beta distribution, after linearly transforming the response variable (i.e., compressing the response range by multiplying it for (sample size −1), adding 0.5, and dividing the sum for the sample size, as suggested by Smithson and Verkuilen, 2006).

Two further datasets allowed us to assess how social tolerance over food varied across groups (Model 3) and individuals (Model 4). In the dataset for Model 3, we entered one data point for each group and session of the social tolerance task (N = 299), specifying the proportion of individuals retrieving food in each session (excluding infants), group size, living conditions, and group identity. We used generalized linear mixed models to assess whether the proportion of eaters differed depending on the group living conditions and/or group size (i.e., including living conditions and group size as test predictors), including group identity as random factor (Model 3). In the dataset for Model 4, we entered one data point for each subject and session number (only excluding infants; *N* = 8,161), specifying the group the subject belonged to, subject’s identity, sex, age class, rank, and centrality, session number, and the proportion of food retrieved by each subject in each session of the social tolerance task. We used generalized linear mixed models to assess whether the proportion of food eaten was predicted by sex, age class, rank, or centrality (i.e., including sex, age class, rank, and centrality as test predictors), when controlling for session number and including subject and group identities as random factors (Model 4). As above, the response variables in both models were transformed and entered in a model with a beta distribution (Smithson and Verkuilen, 2006).

The dataset for Model 5 allowed assessing variation across groups and individuals in neophobia (excluding infants). To ensure that we only included trials in which the stimuli were still novel, for each subject we only included the first six trials of each condition of the neophobia task. We then entered one datum for each of these trials (*N* = 1,334), further specifying the subject retrieving food, whether the food retrieved was familiar (or novel), the individual’s group, living conditions and group size, the individual’s sex, age class, rank, and centrality, trial number (from 1 to 6), condition (i.e., food red, food blue, object yellow, and object silver), proportion of time spent playing (as play behavior may reduce neophobia; see Parker and Gibson, 1977; Lockman, 2000), and proportion of familiar food still available in the testing area when the food was retrieved (as individuals may be “forced” to overcome neophobia when familiar food pieces are limited). We used generalized linear mixed models to assess whether choosing familiar food (as a binomial response) was predicted by the group living conditions and group size, and by the proportion of familiar food available (i.e., including living conditions, group size, and proportion of familiar food available as test predictors). We further controlled for individual’s sex, age class, rank, centrality, proportion of time spent playing, experimental condition, and trial number, including subject and group identities as random factors (Model 5). This model was run with a binomial distribution.

We compared full models (containing test predictors, controls, and random factors) to null models (only containing controls and random factors) by using the function “anova” (Dobson and Barnett, 2018). If there was a significant difference between full and null models, we obtained the *p* values for each predictor, based on likelihood ratio tests (Dobson and Barnett, 2018). We detected no convergence, overdispersion, or collinearity issues (maximum VIFs across models = 4.07) in the models presented.

## Results

### Dominance Style

The study groups varied in their dominance style. Adjusted steepness values decreased as the group size increased, being highest in Cordoba (i.e., 0.203, *N* = 6) and Nürnberg (i.e., 0.202, *N* = 5), while decreasing in Gibraltar (i.e. 0.156, *N* = 19), and being lowest in the two larger groups (i.e., 0.144 in Kintzheim-1, *N* = 59; and 0.136 in Kintzheim-2, *N* = 48). Adjusted steepness values decreased as the group size increased, being highest in Nürnberg (i.e., 0.637, *N* = 5) and Cordoba (i.e., 0.591, *N* = 6), while decreasing in Gibraltar (i.e., 0.225, *N* = 19), and being lowest in the two larger groups (i.e., 0.165 in Kintzheim-1, *N* = 59; and 0.136 in Kintzheim-2, *N* = 48). Similarly, the proportion of agonistic interactions against the hierarchy was lower in the smaller groups (i.e., 1/64 = 2% in Nürnberg; 6/229 = 3% in Cordoba; and 2/125 = 2% in Gibraltar), and higher in the larger ones (i.e., 177/1,412 = 13% in Kintzheim-1 and 199/1,253 = 16% in Kintzheim-2).

### Proportion of Time Spent in Close Spatial Proximity and Grooming Interactions

**Figure 1:**
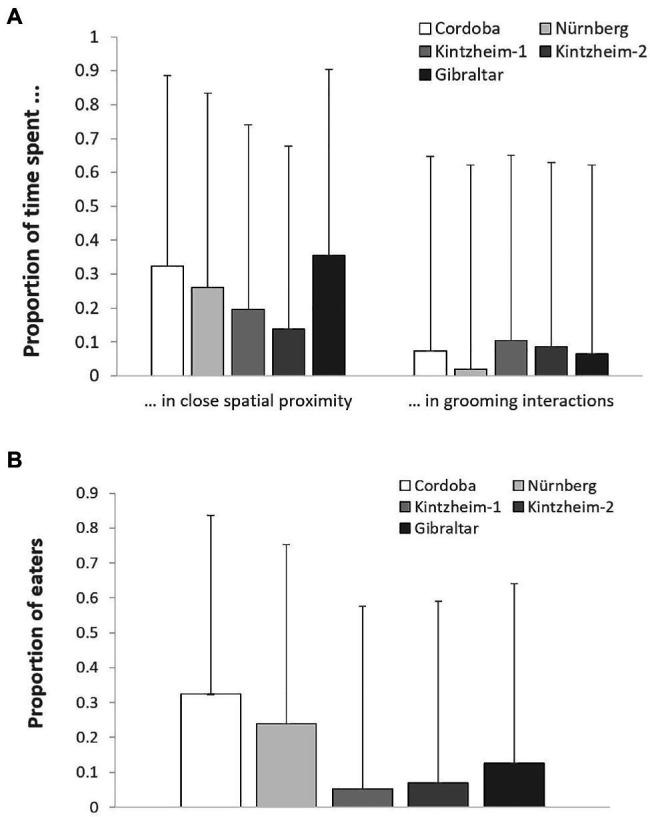
For each study group, **(A)** the mean (+SE) proportion of time spent in close spatial proximity and in grooming interactions, and **(B)** the mean (+SE) proportion of individuals retrieving food in each session of the social tolerance task. Please note that the figure is based on raw data.

The proportion of time spent in close spatial proximity (Model 1) and in grooming interactions (Model 2) varied across study groups depending on their group size (but not living conditions), after controlling for individuals’ sex, age, rank, and social integration. In both cases, the full models significantly differed from the null ones (GLMM, Model 1: χ^2^ = 6.36, df = 2, *p* = 0.042; Model 2: χ^2^ = 8.93, df = 2, *p* = 0.011). In particular, the proportion of time spent in close spatial proximity was significantly higher in smaller groups, while the proportion of time spent in grooming interactions was significantly higher in larger groups (Table 3; Figure 1A).

**Table 3:** Results of Models 1 to 5, including estimates, standard errors (SE), z-values (*z*), and confidence intervals (CIs) for each test and control predictor (in parentheses, the reference category).

Model	Estimate	SE	*z*	2.5% CI	97.5% CI
Model 1: proportion of time spent in close spatial proximity					
Intercept	−1.98	0.43	−4.56	−2.83	−1.13
Living conditions	0.27	0.17	1.56	−0.07	0.60
Group size**	−0.02	0.01	−3.48	−0.03	−0.01
Sex (male)**	−0.26	0.13	−2.02	−0.50	−0.01
*Age (subadult)*	0.51	0.33	1.57	−0.13	1.15
*Rank*	0.37	0.33	1.12	−0.28	1.03
Centrality**	0.87	0.31	2.85	0.27	1.47
Model 2: proportion of time spent in grooming interactions					
Intercept	−3.97	0.45	−8.77	−4.86	−3.09
Living conditions	0.17	0.14	1.25	−0.10	0.45
Group size**	0.02	0.00	5.19	0.01	0.02
Sex (male)**	−0.28	0.13	−2.21	−0.52	−0.03
*Age (subadult)*	−0.16	0.34	−0.46	−0.82	0.51
*Rank*	0.55	0.34	1.62	−0.12	1.23
Centrality**	0.66	0.31	2.12	0.05	1.26
Model 3: proportion of individuals retrieving food in the social tolerance task					
Intercept	−0.48	0.18	−2.73	−0.83	−0.14
Living conditions**	−0.29	0.10	−2.86	−0.49	−0.09
Group size**	−0.03	0.00	−8.59	−0.04	−0.02
Model 4: proportion of food retrieved in the social tolerance task					
Intercept	−2.28	0.12	−19.62	−2.50	−2.05
Sex (male)	0.01	0.03	0.22	−0.06	0.07
Age (juvenile)	0.07	0.07	0.99	−0.06	0.20
Age (subadult)	0.02	0.09	0.26	−0.16	0.20
Rank	0.11	0.09	1.21	−0.07	0.29
Centrality**	0.20	0.09	2.24	0.03	0.38
*Session*	0.00	0.00	0.06	0.00	0.00
Model 5: probability of selecting familiar food in the neophobia task					
Intercept	−3.09	0.95	−3.27	−4.94	−1.24
Living conditions	0.00	0.23	0.00	−0.46	0.46
Group size	0.01	0.01	1.65	0.00	0.03
Proportion familiar food**	4.80	0.50	9.54	3.82	5.79
*Sex (male)*	−0.20	0.32	−0.64	−0.82	0.42
*Age (subadult)*	−1.07	1.03	−1.03	−3.09	0.96
Rank**	1.61	0.79	2.03	0.06	3.17
*Centrality*	0.41	0.68	0.61	−0.91	1.73
*Play proportion*	0.45	0.25	1.83	−0.03	0.94
*Test condition (food2)*	0.20	0.20	1.00	−019	0.59
Test condition (object1)**	−0.45	0.20	−2.24	−0.84	−0.06
Test condition (object2)**	−0.55	0.19	−2.95	−0.92	−0.18
Trial**	−0.05	0.04	−1.20	−0.12	0.03

Group identity was included as a random factor in Models 1 to 5, and subject identity in Models 4 and 5. In Models 1 to 4, responses were proportions and were transformed before running the models. We used a beta distribution for all models, except for Model 5 (where we used a binomial distribution). Control predictors are in italics, and significant test and control predictors are marked by two asterisks.

### Number of Social Partners

The percentage of dyads exchanging grooming at least once (out of all possible dyads among adults and subadults) was highest in the captive groups (in Cordoba and Nürnberg, 8/10=80%), and lowest in the other groups (in Gibraltar: 30/136=22%; in Kintzheim-1: 313/1596=20%; and in Kintzheim-2: 226/861=26%). This means that adults and subadults in Cordoba and Nürnberg (*N*=5 in both groups) interacted, on average, with 1.6 grooming partners, in Gibraltar (*N*=17) with 1.8, in Kintzheim-1 (*N*=57) with 5.5, and in Kintzheim-2 (*N*=42) with 5.4 partners.

### Social Tolerance Over Food

**Figure 2:**
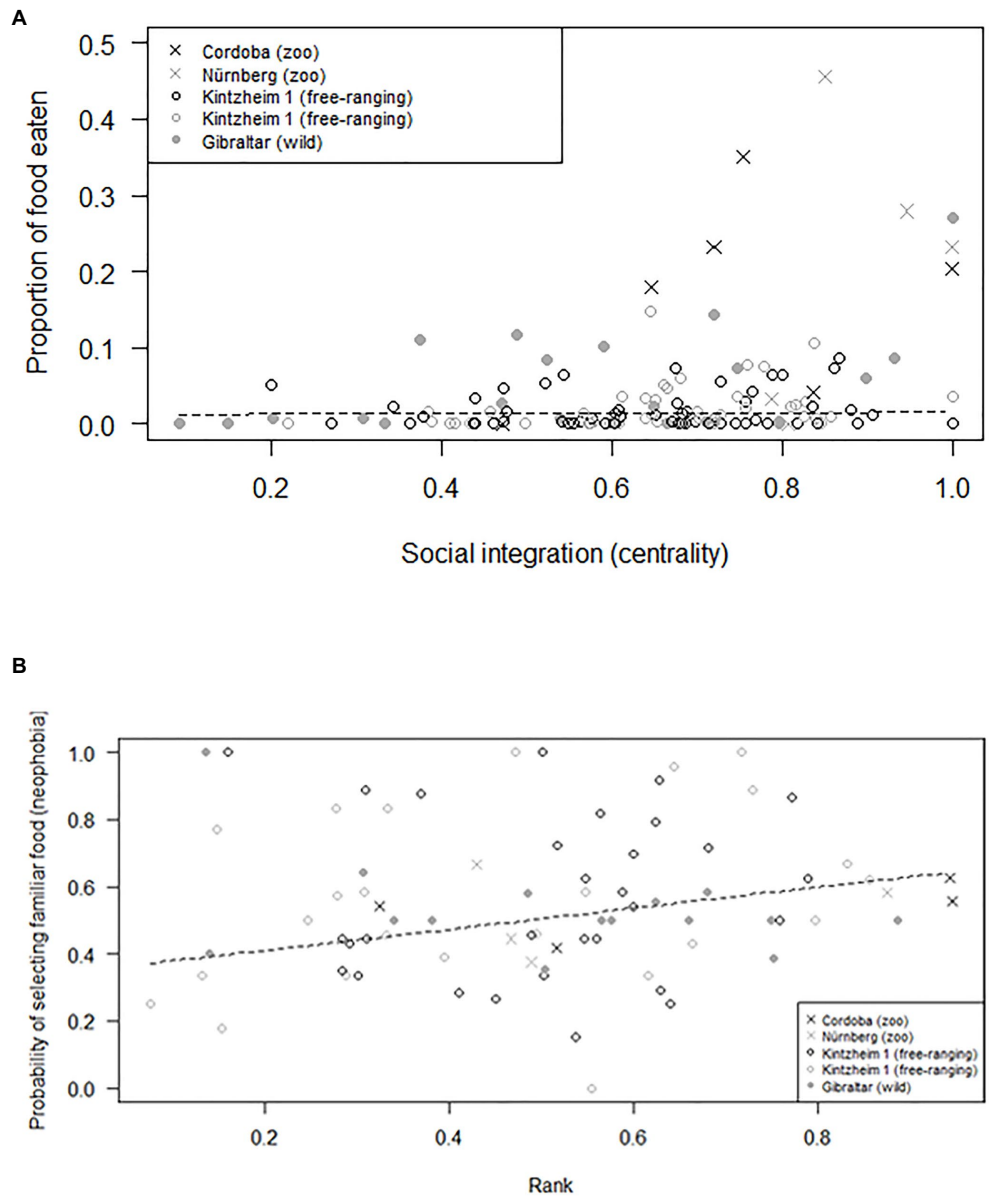
**(A)** Proportion of food retrieved, as a function of individual social integration (measured as eigenvector centrality), and **(B)** probability of selecting familiar food (as a measure of neophobia), as a function of individual rank. Circles and crosses represent individual average response in the social tolerance **(A)** and neophobia **(B)** tasks and are depicted with different symbols depending on the study group they belonged to. The dashed line represents the fitted model, which is like Models 4 **(A)** and 5 **(B)**, but unconditional on all the other predictors that were standardized.

The average number of individuals retrieving food in each session was similar across groups (i.e., 1.2–2.0 in Cordoba and Nürnberg, 2.3 in Gibraltar, 2.5–3.0 in Kintzheim), while the average proportion of individuals eating in each session varied across groups (on average, 0.24–0.33 in Cordoba and Nürnberg, 0.12 in Gibraltar, and 0.04–0.06 in Kintzheim). In particular, the proportion of individuals eating in each session varied across groups, depending on their group size and living conditions. The full model differed significantly from the null model (GLMM, Model 3: χ^2^=16.92, df=2, *p*<0.001). In particular, the proportion of individuals eating in the social tolerance task was significantly higher in smaller groups, and in those living in less natural conditions (Table 3; Figure 1B). Moreover, the individual characteristics of the study subjects predicted the proportion of food they retrieved in the task (GLMM, full-null model comparison for Model 4: χ^2^=11.54, df=5, *p*=0.042). In particular, socially more integrated individuals (i.e., individuals with a higher eigenvector centrality) were significantly more likely to retrieve a higher proportion of food (Table 3; Figure 2A).

### Neophobia

Finally, preference for familiar food (i.e., neophobia) also varied across individuals, but did not significantly vary across groups depending on their group size and living conditions (GLMM, full-null model comparison for Model 5: χ^2^=127.96, df=3, *p*<0.001). In particular, higher-ranking individuals were more neophobic (i.e., they were significantly more likely to retrieve familiar food; Table 3; Figure 2B). However, when the proportion of familiar food still available was higher, all individuals were significantly more likely to choose it (Table 3).

## Discussion

Our results show important behavioral differences across study groups, depending on their group size and living conditions. Individuals in larger groups spent more time in grooming interactions (but not in close spatial proximity) as compared to individuals in smaller groups, partially in line with our predictions (prediction 1a), and in line with previous literature (across primates: Dunbar, 1992; Lehmann et al., 2007). Lehmann et al. (2007), for instance, also found that the time primates invested in grooming interactions increased with group size. By having a higher availability of potential social partners, primates in larger groups may have a higher chance to find suitable partners and/or may have a higher need to invest time in grooming to maintain group cohesiveness (see Dunbar, 1992; Lehmann et al., 2007; Majolo et al., 2008), although they may need to compromise on their grooming time when groups became too large (Lehmann et al., 2007). In line with this, groups also differed in the average number of grooming partners per individual, which was on average much higher in larger than in smaller groups (i.e., 5.4–5.5 in the two larger groups, versus 1.6–1.8 in the three smaller ones). However, the proportion of dyads engaging in grooming interactions at least once was higher in the two smaller captive groups, where 80% of the potential dyads engaged in grooming (in contrast to 20–26% in the other groups). These results suggest that larger groups may offer individuals a wider range of potential partners, so that individuals can increase the average number of grooming partners, while becoming more selective in their choice (i.e., engaging in grooming interactions with only around 20% of the group members; also see Silk et al., 1999, on baboons, and Berman and Thierry, 2010, on macaques). If individuals were simply grooming as many partners as possible, the average number of grooming partners should have been higher in smaller groups. These results are in line with a former study by Berman and Thierry (2010) showing that when the number of potential partners increased, female Tonkean macaques (*Macaca tonkeana*) spent a similar amount of time in grooming interactions, but focused their grooming on a smaller subset of partners, becoming more selective. Furthermore, in contrast to our predictions, larger groups did not spend more time in spatial proximity (prediction 1a), nor living conditions affected the time spent in proximity or grooming interactions (prediction 2a).

Individuals in larger groups had less despotic dominance styles, in line with our predictions (prediction 1b). Smaller groups had higher steepness values and a lower proportion of agonistic interactions against the hierarchy—two measures linked to more despotic dominance styles (Thierry, 2000, 2007; Young and Isbell, 2002; de Vries et al., 2006; Thierry et al., 2008). These findings raise interesting questions on the potential mechanisms at work. Probably, higher-ranking individuals in larger groups are less successful at exerting control over the other group members and at maintaining linear hierarchies, with the result that larger groups are less despotic (Mori, 1977; Zhang and Watanabe, 2014; but see Yamagiwa and Hill, 1998). However, it is also possible that when fewer members are available to populate all the rank “positions” along the rank continuum, group members act in accordance with an absolute and not a relative scale of dominance. In line with this, larger dominance distance between rank-adjacent animals decreased the probability of agonistic interactions against the hierarchy; reinforcing the idea these behaviors present a higher risk of disrupting the social homeostasis of the group. Crucially, these findings suggest that dominance style, which is usually considered to be species-specific (macaques: Thierry, 2000, 2007; Flack and de Waal, 2004; Thierry et al., 2004), may also importantly vary across conspecific groups, for instance, in terms of frequency and intensity of aggression, frequencies of counter-aggression, and grooming patterns (Japanese macaques: Zhang and Watanabe, 2014). This has important implications for studies relying on observations of captive individuals, because groups in captivity are often much smaller and might thus show more despotic dominance styles than their wild counterparts, so that generalizations may be problematic. In contrast to our predictions (prediction 2b), however, living conditions did not explain differences across study groups.

Groups living in less natural conditions were more tolerant over food, in line with our predictions (prediction 2c). In captive groups, individuals were completely food provisioned and might have thus faced limited food competition, leading to a general increase in tolerance over food, and/or to a decrease in the intrinsic value of food (Janson and van Schaik, 1988; Isbell, 1991). However, in contrast to our prediction 1c, larger groups did not have higher tolerance levels. Instead, the proportion of individuals retrieving food was higher in smaller groups. This may appear surprising, as higher-ranking individuals in smaller groups should be able to better monopolize resources (see Gomez-Melara et al., 2021, in macaques). However, it is also possible that individuals may tolerate (or may not manage to displace) a specific number of partners in the testing area, so that in smaller groups the *proportion* of individuals retrieving food would appear higher as a “side effect.” Indeed, while the average proportion of individuals retrieving food strongly varied across groups, the average number of individuals retrieving food was more similar across groups. At first sight, this might suggest that, after all, tolerance over food did not vary across groups, as a similar number of individuals could access food. However, one should recall that the food provided in this task was proportional to the number of adults in each group (i.e., there was approximately one piece of food available for every second individual, in all groups). Therefore, a similar number of individuals retrieving food across study groups suggests a higher monopolization of food in larger groups, as (proportionally) fewer individuals could gain access to food, obtaining more food pieces. Therefore, although the absolute number of group members tolerated in a feeding context was similar across groups, the distribution of resources was not. Finally, it is also possible that food has a lower intrinsic value in captive groups that are completely food provisioned, so that monkeys may be less willing to engage in aggressive interactions to access it.

Beyond differences across groups, we found inter-individual differences in access to food. While sex, age, and rank did not affect the proportion of food retrieved, social integration (i.e., eigenvector centrality) did. Highly gregarious individuals and/or those connected to highly gregarious partners (Farine and Whitehead, 2015) were more likely to retrieve food. The link between social integration and access to food has not been often investigated, but new studies converge in showing a reliable effect of social integration on the likelihood to access food. In a previous study comparing the Gibraltar group to other three wild groups of macaques with different dominance styles (i.e., Japanese macaques, long-tailed macaques, *Macaca fascicularis*, and moor macaques, *M. maura*), for instance, we found that more central individuals had a higher probability of retrieving food in all species (Amici et al., 2020). Such link between sociality and access to food has also been found in other species (Guinea baboons, *Papio papio*: Dell’Anna et al., 2019) and suggests that social integration, by affecting individuals’ ability to access resources, might have a strong direct impact on their fitness and well-being (Dell’Anna et al., 2019; Amici et al., 2020). These results are also in line with previous studies showing a link between sociality and fitness in both human (Smith and Christakis, 2008; Holt-Lunstad et al., 2010) and non-human primates (e.g., baboons: Silk et al., 2003, 2009, 2010; Archie et al., 2014; Assamese macaques, *Macaca assamensis*: Schülke et al., 2010), including Barbary macaques (McFarland and Majolo, 2013; Lehmann et al., 2016). However, our findings further suggest that the fitness benefits provided by sociality may be more direct than previously thought, with higher social integration directly providing benefits in terms of access to resources, likely through increased tolerance at food sites and higher sharing of resources.

In contrast to our expectations, individuals in larger groups (prediction 1d) and living in less natural conditions (prediction 2d) did not show lower levels of neophobia, although there was variation across individuals. Higher-ranking individuals preferentially ate familiar over novel food in all groups, also when controlling for the proportion of familiar food still available. This is in line with the previous literature suggesting that higher-ranking individuals, by generally having better access to resources, might gain lower payoffs from novelty and thus be more neophobic (Wolf et al., 2007; in birds: Hegner, 1985; Greenberg-Cohen et al., 1994; Lahti, 1998; Greenberg and Mettke-Hofmann, 2001; in fish: Laland and Reader, 1999). In social species, in particular, more dominant individuals usually have better access to resources like space, food, or mates and may thus be less prone to explore novel resources than subordinates (Greenberg and Mettke-Hofmann, 2001; Wolf et al., 2007). However, when the proportion of familiar food still available was higher, all individuals were more likely to prefer familiar food. This suggests that lower-ranking individuals, rather than simply being less neophobic, were more likely to overcome their neophobic tendencies than higher-ranking individuals, as they likely have less choice in terms of food that can be accessed without risk of aggression. Therefore, although neophobia is usually considered a personality trait that remains rather constant throughout life history, social contingencies may determine how likely this trait is displayed in different contexts (see Greenberg and Mettke-Hofmann, 2001; Greenberg, 2003; Mettke-Hofmann, 2007).

Overall, our results confirm that intra-specific variation is an important aspect of primate behavior and suggest that special caution should be taken when generalizing results across conspecific populations (see, e.g., Strier, 2016). Because of resource constraints, researchers in comparative psychology are often forced to collect data on limited number of study subjects when comparing species, which reduces the possibility to effectively assess intra-specific variation (Strier, 2016; van Leeuwen et al., 2018). Still, conspecific groups with different socio-ecological characteristics might show very different behaviors. Our results, however, further suggest that social factors like group size may have a stronger impact on primate behavior, as compared to the living conditions of the study groups. In our study, group size affected the number of grooming partners, their dominance style, social interactions, and tolerance over food. In contrast, living conditions were only linked to differences in tolerance levels. This suggests that while generalizations should always be taken with caution, generalizing observations of captive (or semi-free-ranging) individuals to wild ones does not necessarily posit more problems than doing that across conspecific wild groups, at least if captive groups have natural socio-demographic characteristics and do not live in socio-ecologically deprived environments (for a further discussion about it, see Boesch, 2007; Tomasello and Call, 2008).

Our study has various potential limitations. First, we only included five groups, which did not allow us to optimally measure variation in group size and living conditions, especially considering that these variables are often correlated with each other (e.g., in zoos, groups are usually smaller, completely food-provisioned and with limited freedom of movement), and that behavior can both affect and be affected by socio-ecological and demographic conditions (Strier, 2011). Moreover, some of the study groups, especially those in captivity, were rather small, so that inter-individual variation was limited (e.g., we only had few captive males in our study groups). Thus, the specific group composition might have affected the behavioral patterns observed, as social interactions can differ across individuals depending on their demographic characteristics. Even though our models accounted for inter-individual variation, future studies should aim to collect data on larger groups. Second, our observational effort was limited and unevenly distributed across study groups, although there may be temporal variation in several of the variables we assessed (e.g., seasonal variation in social behavior). Future studies should account for this by including longer behavioral observations that ideally include more seasons. However, we do not think that our limited observational effort can explain our findings. The two free-ranging groups, for instance, were tested in two different periods, and so were the two captive groups. Nonetheless, these groups behaved in a similar way, in our analyses, suggesting that intra-specific variation in terms of, for example, living conditions, might be stronger than seasonal variation. Similarly, future studies should better control for temporal variation in individuals’ response during neophobia and social tolerance tasks, which might depend on differences in motivation also linked to the stimuli used. Furthermore, future studies should ideally include more genera, as different socio-ecological characteristics and living conditions may not affect all species in the same way. Finally, it will also be important to further assess how different aspects of tolerance over food (e.g., absolute number of group members tolerated in proximity in a feeding context, distribution of food resources across individuals) vary in primates depending on their group size. Although researchers are well aware of the exceptional behavioral diversity shown by primates (e.g., Struhsaker, 2000; Strier, 2003, 2016; Mitani et al., 2012), the “source” of such diversity is still to be fully understood.

## Data Availability Statement

The raw data supporting the conclusions of this article will be made available by the authors, without undue reservation.

## Ethics Statement

The animal study was reviewed and approved by Cordoba Zoo (Spain), Nürnberg Zoo (Germany), La Montagne des Singes (France), and Helping Hand Trust (Gibraltar). The study was carried out in accordance with the national regulations of all the countries in which the study was conducted.

## Author Contributions

FA, AW, and BM designed the research, with extensive feedback by LF on the methodological part. AC collected data. FA analyzed the data and wrote the paper, with extensive input from all authors. All authors contributed to the article and approved the submitted version.

## Funding

FA was financed by a German Research Foundation (DFG) research grant (AM 409/4–1) during this study.

## Conflict of Interest

The authors declare that the research was conducted in the absence of any commercial or financial relationships that could be construed as a potential conflict of interest.

## Publisher’s note

All claims expressed in this article are solely those of the authors and do not necessarily represent those of their affiliated organizations, or those of the publisher, the editors and the reviewers. Any product that may be evaluated in this article, or claim that may be made by its manufacturer, is not guaranteed or endorsed by the publisher.
