# Corrigendum: Intra-specific variation in the social behavior of Barbary macaques (*Macaca sylvanus*)

**DOI:** 10.3389/fpsyg.2023.1192731

**Published:** 2023-05-02

**Authors:** Federica Amici, Anja Widdig, Lorenzo von Fersen, Alvaro Lopez Caicoya, Bonaventura Majolo

**Affiliations:** ^1^Department of Human Behavior, Ecology and Culture, Research Group “Primate Behavioural Ecology”, Max Planck Institute for Evolutionary Anthropology, Leipzig, Germany; ^2^Faculty of Life Science, Behavioral Ecology Research Group, Institute of Biology, University of Leipzig, Leipzig, Germany; ^3^Zoo Nuernberg, Nuernberg, Germany; ^4^Department of Clinical Psychology and Psychobiology, Faculty of Psychology, University of Barcelona, Barcelona, Spain; ^5^Institute of Neurosciences, University of Barcelona, Barcelona, Spain; ^6^School of Psychology, University of Lincoln, Lincoln, United Kingdom

**Keywords:** intra-specific variation, Barbary macaques, neophobia, social integration, access to food, social behavior

In the published article, there was an error. It was stated that the adjusted steepness values of our study groups were 0.637 in Nürnberg, 0.591 in Cordoba, 0.225 in Gibraltar, 0.165 in Kinztheim-1 and 0.136 in Kintzheim-2. However, there was a mistake in the script used to calculate adjusted steepness. After correcting the mistake in the script, the correct adjusted steepness values are: 0.202 in Nürnberg, 0.203 in Cordoba, 0.156 in Gibraltar, 0.144 in Kinztheim-1 and 0.136 in Kintzheim-2. A correction has been made to *Results, Dominance Style, Paragraph 1*. The corrected paragraph is shown below.

“The study groups varied in their dominance style. Adjusted steepness values decreased as the group size increased, being highest in Cordoba (i.e., 0.203, *N* = 6) and Nürnberg (i.e., 0.202, *N* = 5), while decreasing in Gibraltar (i.e. 0.156, *N* = 19), and being lowest in the two larger groups (i.e., 0.144 in Kintzheim-1, *N* = 59; and 0.136 in Kintzheim-2, *N* = 48). Adjusted steepness values decreased as the group size increased, being highest in Nürnberg (i.e., 0.637, *N* = 5) and Cordoba (i.e., 0.591, *N* = 6), while decreasing in Gibraltar (i.e., 0.225, *N* = 19), and being lowest in the two larger groups (i.e., 0.165 in Kintzheim-1, *N* = 59; and 0.136 in Kintzheim-2, *N* = 48). Similarly, the proportion of agonistic interactions against the hierarchy was lower in the smaller groups (i.e., 1/64 = 2% in Nürnberg; 6/229 = 3% in Cordoba; and 2/125 = 2% in Gibraltar), and higher in the larger ones (i.e., 177/1,412 = 13% in Kintzheim-1 and 199/1,253 = 16% in Kintzheim-2).”

Please note that the steepness values presented in the paper were only given for descriptive reasons and these steepness values were not included in any further analysis. Thus, the results of our statistical analyses in the paper are not affected by this mistake. Moreover, the changes in the steepness values do not affect the order of the steepness values for the various study groups: the correct steepness values remain highest in Nürnberg and Cordoba, while decreasing in Gibraltar, and being lowest in the two larger groups (i.e., Kintzheim-1 and Kintzheim-2). Therefore, this mistake does not affect the interpretation of our results and our conclusions.

The authors apologize for this error and state that this does not change the scientific conclusions of the article in any way. The original article has been updated.

